# Osteosarcopenia: Prevalence and 10-Year Fracture and Mortality Risk – A Longitudinal, Population-Based Study of 75-Year-Old Women

**DOI:** 10.1007/s00223-023-01181-1

**Published:** 2024-02-01

**Authors:** Tine Kolenda Paulin, Linnea Malmgren, Fiona E McGuigan, Kristina E Akesson

**Affiliations:** 1 Department of Clinical Sciences Malmö, Clinical and Molecular Osteoporosis Research Unit, Lund University, Malmö, Sweden; 2 Department of Geriatrics, Skåne University Hospital, Malmö, Sweden; 3 Department of Orthopaedics, Skåne University Hospital, Malmö, Sweden

**Keywords:** Osteosarcopenia, Osteoporotic fracture, Mortality, Older individuals

## Abstract

Osteosarcopenia is the coexistence of low bone mass and sarcopenia. In older women, its prevalence is not well described, and it is unknown if sarcopenia is additive to low bone mass for fracture and mortality risk. The study investigated prevalence of osteosarcopenia and if osteosarcopenia is associated with higher fracture and mortality risk than low bone mass alone in older community-dwelling women. The longitudinal, population-based OPRA Cohort (*n* = 1044), all aged 75 at inclusion, followed for 10 years. Using WHO and EWGSOP2 definitions for low bone mass (T-score < −1.0 femoral neck) and sarcopenia (knee strength; appendicular lean muscle mass) women were categorized (1) Normal, (2) Low bone mass (LBM), and 3) Osteosarcopenia (probable; confirmed). Risk of hip, major osteoporotic fracture, and mortality were estimated. Osteosarcopenia_confirmed_ prevalence increased from age 75 to 80 and 85 from 3.0% (29/970) to 4.9% (32/656) to 9.2% (33/358) but prevalence is potentially 2–4 times higher (11.8%, 13.4%, 20.3%) based on osteosarcopenia_probable_. Having osteosarcopenia_probable_ significantly increased 10-year risk of hip fracture (HR_adj_ 2.67 [1.34–5.32]), major osteoporotic fracture (HR_adj_ 2.04 [1.27–3.27]), and mortality (HR_adj_ 1.91 [1.21–3.04]). In contrast, LBM increased osteoporotic fracture risk (HR_adj_ 2.08 [1.46–2.97], but not hip fracture (HR_adj_ 1.62 [0.92–2.85]) or mortality (HR_adj_ 0.94 [0.64–1.38]). Median time-to-hip fracture was 7.6 years (normal), 6.0 years (LBM), and 5.7 years (osteosarcopenia_probable_). Prevalence of confirmed osteosarcopenia is almost 10% at age 85. Probable osteosarcopenia significantly increased risk of hip and major osteoporotic fractures and mortality more so than low bone mass alone.

**Supplementary Information:**

The online version contains supplementary material available at 10.1007/s00223-023-01181-1.

## Introduction

With the proportion of older individuals increasing rapidly worldwide [1], the need to promote healthy aging is essential, in order to prolong the number of healthy years alive. Age-related diseases associated with decline in the musculoskeletal system include osteoporosis and sarcopenia [2, 3]. The consequences of these include falls and fractures, which in turn leads to hospitalization, immobilization, frailty, institutionalization, and death [4]. Apart from the personal suffering, the magnitude of the socio-economic cost is enormous [5] and expected to increase further within the coming years.

Osteoporosis results from low bone mass and micro-architectural deterioration, which decreases strength and increases the risk of fragility fractures. The progressive deterioration of skeletal muscle, sarcopenia, is characterized by low muscle mass and strength, resulting in decreased muscle function, which contributes to increased risk of falls [6]. The combination of having both conditions, osteosarcopenia (OS), is a relatively new term, originally described in 2009 to emphasize the interaction between bone and muscle and the impact on fracture [7]. By this, the two conditions are visualized in a new context with their coexistence possibly pushing an older individual into a vicious spiral leading to loss of independence.

The pathophysiology of osteosarcopenia includes mechanical, biochemical, genetic, and lifestyle factors, such as alcohol, smoking, physical activity, and diet [3, 6, 8]. The oldest theory of the interaction between muscle and bone is the mechanostat hypothesis which describes, according to Wolff’s law, how bone remodeling is a consequence of mechanical (muscle) loading [3, 6]. The biochemical linkage between the two tissues, though less well defined, includes sex hormones and active substances originating in muscle (myokines) and bone (osteokines) [8].

Since its inception, interest in osteosarcopenia has accelerated with studies trying to elucidate its prevalence among older individuals and to understand the consequences and impact on musculoskeletal health. Although, reports vary above the age of 60 prevalence estimates range from 1.5% to 34% [9, 10] in the general population. The wide range is due to differences in the populations studied, study design, and the methodology employed. Among those who have fractured, the highest rates have been observed (28.7% to 65.7%) [10] highlighting the linkage between these conditions.

However, a major challenge has been to find the most suitable definition of osteosarcopenia [6, 8] and various definitions of “osteo” and “sarcopenia” have been applied, making comparisons between studies difficult [2, 8]. While some studies include only “osteoporosis” [7, 8, 11], others also include “osteopenia” [9, 10, 12–15]. As for sarcopenia, the two most widely used definitions come from the revised editions of the European Working Group on Sarcopenia in Older People 2 (EWGSOP2) and the Asian Working Group for Sarcopenia (AWGS 2019). Although both are based on muscle strength, muscle composition (quantity or quality), and physical performance, different combinations of these are used to describe probable sarcopenia, confirmed sarcopenia, and severe sarcopenia [16, 17].

Based on this, several gaps in knowledge have been identified. First, since the majority of existing studies are cross-sectional, include both men and women, and are performed in sub-populations [2], the true age-related *prevalence* of osteosarcopenia is still not known. Secondly, while several studies have investigated the relationship between osteosarcopenia, falls, fracture, and mortality, it remains unclear if the *risk* is worsened by the coexistence of both conditions compared to low bone mass alone [9, 12, 13, 15, 18].

The present study is a longitudinal investigation of osteosarcopenia in the OPRA cohort of community-dwelling older women, all aged 75 at inclusion. The primary aim is to determine the prevalence of osteosarcopenia at ages 75, 80, and 85, and the secondary aim is to examine the association between osteosarcopenia, fracture, and mortality.

## Materials and Methods

### Study Sample

The women in this study are from the population-based Osteoporosis Prospective Risk Assessment (OPRA) cohort in Malmö, Sweden, previously described in detail [19, 20]. A total of 1604 community-dwelling older women were randomly selected from the city register from 1995 to 1999 and invited by letter at their 75th birthday. No exclusion criteria were applied. The cohort was originally designed to study fracture, and the age at inclusion was chosen to capture the greatest number of fractures.

At baseline, 1044 women agreed to participate in the investigations (65% response rate). Reasons for non-attendance were illness (*n* = 152), unwillingness (*n* = 376), or non-responder despite several attempts to be reached (*n* = 32). Follow-up visits were performed after 5 years (age 80, *n* = 715) and 10 years (age 85, *n* = 382). Reasons for not attending follow-up at age 80 included dead (*n* = 96), illness (*n* = 72), no reason (*n* = 130) and other explanations (*n* = 31). Reasons for not attending follow-up at age 85 included dead (*n* = 307), illness (*n* = 140), no reason (*n* = 158), and other explanations (*n* = 57) [20]. The women were followed until October 2012 for fractures and mortality.

Extensive investigations were performed at all visits including physical measurements and tests, detailed questionnaires (on medications, nutrition, lifestyle, smoking, alcohol habits, diseases, mobility, etc.), and blood samples were collected. The study was approved by the Regional Ethics Committee in Lund (Dnr: 2014804) and in accordance with the Helsinki declaration. All participants provided written informed consent.

### Musculoskeletal Composition

Areal bone mineral density (BMD, g/cm^2^) of the femoral neck and whole-body lean muscle mass (kg) were measured using the same dual-energy x-ray absorptiometry (DXA) machine (Lunar DPX-L, GE Lunar, Madison, WI) throughout the study at Skåne University Hospital, Malmö. Calibrations were performed daily using a phantom supplied by the manufacturer. Precision was determined by duplicate measurements on 30 OPRA participants and 31 measurements of the spine phantom. Coefficient of variation was 4.0% for femoral neck BMD and 10.6% for whole-body lean mass. No drifts in phantom measurements were observed [21, 22].

Based on DXA measured lean mass, appendicular lean muscle mass (ALM) was calculated as the sum of lean muscle mass of the arms plus legs, divided by height squared (ALM/height^2^, kg/m^2^) as recommended by European Working Group on Sarcopenia in Older People (EWGSOP2) [16]. Low muscle mass was defined as ALM < 5.5 kg/m^2^ [16], based on the reference range of a healthy Australian cohort based on −2 SD [23].

Muscle strength was measured as isometric torque of the lower limb (knee strength) as handgrip strength measurements were not available at baseline. Knee strength was measured as maximal knee extension isometric contraction at 90˚ (Newton meter seconds, Nms) using a computerized isokinetic dynamometer (Biodex Medical Systems, version 4.5.0., Biodex Corporation, Shirley, New York). The best out of three measurements of the dominant leg, each lasting 5 s, was recorded. Low muscle strength was defined as < 175 Nms, equating to a handgrip strength of < 16 kg, based on detailed calculations described elsewhere [24].

### Definition of Low Bone Mass, Sarcopenia, Osteosarcopenia, and Reference Group

Low bone mass (LBM) was defined as T-score < −1.0 at the femoral neck to capture both osteopenia (−2.5 SD < T-score < −1 SD) and osteoporosis (T-score ≤ −2.5 SD) according to the World Health Organization (WHO) classification [25]. We included both osteopenia and osteoporosis, since the majority of women who suffer a fracture do not have a BMD reaching the threshold of osteoporosis; to maximize sample size and to facilitate comparison with the existing literature.

Using the consensus guidelines of the EWGSOP revised edition (2019) [16], confirmed sarcopenia was defined as low muscle strength plus low muscle mass. Probable sarcopenia was defined as low muscle strength alone.

In this study, for estimating prevalence, we define confirmed osteosarcopenia as low bone mass plus confirmed sarcopenia to facilitate comparison with the existing literature, while for completeness, we additionally report estimates based on also including probable sarcopenia.

In all other analysis, to maximize sample size, we define probable osteosarcopenia as low bone mass plus confirmed OR probable sarcopenia.

The normal (reference) group was defined as those having normal bone mass (T-score ≥ − 1) *plus* normal muscle parameters (knee strength ≥ 175 Nms *and* muscle mass ≥ 5.5 kg/m^2^).

To estimate prevalence at baseline, 5-year, and 10-year follow-up, women were dichotomised as ‘having osteosarcopenia or not.’ To label an individual as having osteosarcopenia required data for 2 or 3 variables depending on use of probable or confirmed osteosarcopenia as the definition, while 1 variable could be sufficient to assign them as ‘not’. Data were available for 970, 656 and 358 women at respective visits.

To estimate fracture and mortality risk, the women were categorized into three groups, which required available baseline data for all 3 variables. The groups were (1) normal, *n* = 170, (2) low bone mass, *n* = 489, and (3) osteosarcopenia_probable_, *n* = 99 (Supplementary Table 1).

### Falls, Frailty, and Impaired Mobility

Information about falls sustained within the previous 12 months was obtained through questionnaires.

A frailty index constructed according to the principles of Searle et al. [26] was available at all visits. The index ranges between 0.0 and 1.0; the higher the score, the frailer the individual. Full details of the index and its construction are described elsewhere [27]. Frailty index was analyzed as a continuous variable and additionally using the empirical cut-off of > 0.25 to define those who were frail [28, 29]. Mobility was described using an ADL score of 8 levels of mobility transformed to a dichotomous score with the cut-off ‘walking ability with or without device/help’. Impaired mobility was defined as requiring a walking aid, being unable to walk without personal support or being bedbound.

### Hip and Major Osteoporotic Fracture

Incident fractures data were collected through continuous search by personal identification number on x-ray files from the Radiology Department, Malmö, Skåne University Hospital (October 2012). As the Orthopedic Department was the only unit handling fractures in the capture area, the information loss was low [30]. Major osteoporotic fracture was defined as a fracture of the proximal humerus, distal radius, vertebra, hip, or pelvis. Pathological and high energy fractures were excluded.

### Mortality

Information on incident deaths were retrieved from the Swedish National Population Register (October 2012).

## Statistics

Descriptive data were reported as mean with standard deviations (SD) or medians with interquartile range (IQR), as appropriate for continuous variables, and as number with percentage for categorical variables.

In all analyses apart from estimation of prevalence, comparisons  were made between three groups: “Normal,” “Low bone mass,” and “Probable osteosarcopenia”.

Differences between the three groups at baseline were estimated using one-way ANOVA (with post hoc analysis) on normally distributed data. Non-parametric tests were used in case of non-normally distributed data or Pearson Chi-square test. Cox proportional hazard models, unadjusted and adjusted for s-25(OH)D3, alcohol and polypharmacy (3 or more medications) in fracture analyses and for smoking, alcohol, polypharmacy, albumin, and CRP in mortality analyses were performed to calculate 10-year risk for a first hip fracture, osteoporotic fracture, and death in the low bone mass and probable osteosarcopenia groups (normal group as reference). The variables adjusted for were determined using Directed Acyclic Graph (DAGs) [31]. Kaplan–Meier curves were performed to depict observed time to first hip fracture, osteoporotic fracture, and mortality in the three groups. *p*-value for difference was calculated using log-rank test.

The data presented are secondary exploratory analyses, hence power calculations are not stated. A priori power analyses prior to collection of the cohort have been reported previously [32].

All statistical analyses were performed using SPSS (IBM Corp. Released 2021. IBM SPSS Statistics for Windows, Version 28.0. Armonk, NY: IBM Corp). A *p*-value < 0.05 was considered nominally significant.

## Results

Each of the 29 women with confirmed sarcopenia (i.e., low muscle strength plus low muscle mass) had concurrent low bone mass. Conversely, of those women with low bone mass, only 4% (9/733) also had confirmed sarcopenia. While no one with confirmed sarcopenia had normal bone mass, seventeen women had normal bone mass together with one of the criteria for sarcopenia (low muscle strength or mass).

The prevalence of confirmed osteosarcopenia in these community-dwelling women increased from 3.0% (29/970) at age 75 (baseline) to 4.9% (32/656) at age 80 and 9.2% (33/358) at age 85. Using the less stringent, probable osteosarcopenia definition, prevalence is 2 to 4 times higher, an estimated 11.8% (106/896), 13.4% (71/531), and 20.3% (59/290).

Characteristics of the normal, low bone mass (LBM), and osteosarcopenia_probable_ groups at baseline (age 75) are shown in Table 1**.** Among the low bone mass group, one-third (34.6%, 169/489) had osteoporosis. Within the osteosarcopenia_probable_ group, almost half (44.4%, 44/99) had osteoporosis with the remaining having osteopenia.

**Table 1:** Baseline characteristics of the OPRA cohort categorized by the groups: normal, low bone mass (LBM), and probable osteosarcopenia (OS_prob_)

									Comparisons		
	ALL *n* = 758		Normal *n* = 170		Low Bone Mass *n* = 489		OS_prob_ *n* = 99		OS_prob_ vs Normal	OS_prob_ vs LBM	LBM vs Normal
Variable	Mean	(SD)	Mean	(SD)	Mean	(SD)	Mean	(SD)	*p*-value	*p*-value	*p*-value
Age (years)	75.2	(0.14)	75.2	(0.14)	75.2	(0.14)	75.2	(0.16)	–	–	–
Weight (kg)^*^	67.0	(13)	73.0	(12)	65.0	(13)	63.0	(15)	< 0.001	0.004	< 0.001
Height (cm)	160.6	(5.7)	162.8	(5.5)	160.3	(5.5)	157.9	(5.7)	< 0.001	< 0.001	< 0.001
BMI (kg/m^2^)	26.2	(3.6)	27.9	(3.3)	25.8	(3.2)	25.2	(5.0)	< 0.001	0.553	< 0.001
BMD, fem neck (g/cm^2^)^*^	0.750	(0.2)	0.942	(0.1)	0.717	(0.1)	0.692	(0.2)	< 0.001	0.098	< 0.001
T-score, fem neck (SD)^*^	−1.9	(1.5)	−0.3	(0.8)	−2.2	(1.1)	−2.4	(1.3)	< 0.001	0.098	< 0.001
Muscle mass (ALM, kg/m^2^)^a^	6.2	(0.8)^*^	6.5	(0.8)^*^	6.2	(0.7)^*^	6.0	(0.8)	< 0.001	0.002	< 0.001
Muscle strength, (Nms)^b^	277.8	(106)^*^	304.9	(61.5)	284.8	(88)^*^	149.1	(51)^*^	< 0.001	< 0.001	0.013
Gait speed (m/s)	1.23	(0.3)	1.29	(0.2)	1.27	(0.2)	0.91	(0.5)^*^	< 0.001	< 0.001	0.603
Frailty index^*c^	0.15	(0.1)	0.14	(0.1)	0.14	(0.1)	0.23	(0.2)	< 0.001	< 0.001	0.862
p-CRP^*^ (mg/L)^*^	1.7	(2.6)	2.0	(2.7)	1.6	(2.4)	2.0	(3.4)	0.938		0.041
p-albumin (g/L)	40.0	(3)*	40.0	(3)*	40.2	(2.5)	40.0	(3.0)^*^	0.985		0.846
s-25(OH)D3 (nmol/L)	61.0	(26)^*^	59.0	(28)^*^	62.0	(26)^*^	60.9	(20.4)	0.562		0.053
p-creatinine (µmol/L)^*^	73.0	(17)	75	(16)	73	(16)	71	(22)	0.678		0.114
p-cystatin C (mg/L)^*^	1.04	(0.3)	1.06	(0.3)	1.02	(0.3)	1.09	(0.5)	0.169	0.010	0.217
eGFR cysC (mL/min/1.73m^2^)	65.0	(17.0)	64.5	(16.4)	66.1	(16.4)	60.3	(20.1)	0.148	0.007	0.656
Smoking, pack years^*^	20.0	(19)	21.1	(25)	17.5	(20)	22.8	(12)	0.772	0.146	0.264
										–	
	*n* (%)		*n* (%)		*n* (%)		*n* (%)		*p* (chi)		
Current smoker	103	(13.8)	12	(7.2)	72	(14.8)	19	(19.6)	0.01		
Alcohol abstainer	131	(17.4)	28	(16.7)	75	(15.5)	28	(28.6)	0.027		
Severe disease/tumor	115	(15.5)	27	(16.2)	76	(15.9)	12	(12.6)	0.704		
Polypharmacy^d^	317	(41.8)	73	(42.9)	193	(39.5)	51	(51.5)	0.081		
Fallen in the last year	177	(27)	38	(26)	100	(24)	39	(43)	0.001		
Impaired mobility	44	(5.8)	4	(2.4)	19	(3.9)	21	(21.2)	< 0.001		
Frail individuals^e^	138	(18.2)	30	(17.6)	68	(13.9)	40	(40.4)	< 0.001		

^*^Median (IQR); ^a^Appendicular lean Muscle mass; ^b^Knee strength (maximal knee extension isometric contraction at 90°); ^c^Frailty index (cut-off range 0.0–1.0 on each variable); ^d^Polypharmacy (≥ 3 medications); and ^e^Frail status (frailty index ≥ 0.25). Probable osteosarcopenia (low bone mass plus confirmed sarcopenia or probable sarcopenia)

### Characteristics of the Probable Osteosarcopenia Group

The osteosarcopenia_probable_ group had, as expected, lower muscle strength and muscle mass, in particular muscle strength, which was only half of that in the normal group (149.1 (51) vs 304 (61.5), p < 0.001) (Table 1). Correspondingly, gait speed was reduced and impaired mobility was more prevalent compared to both other groups – nearly ten times higher than in the normal group (21.2% vs 2.4%) and more than five times higher compared to the LBM group (21.2% vs 3.9%). Almost double the number had fallen in the previous 12 months, compared to the normal group (43% vs 26%) and even in comparison to the low bone mass group (43% vs 24%). Frailty index was also higher (0.23 vs 0.14 for both; p < 0.001) and more than twice as many were defined as frail (40.4%).

### Risk of Hip and Major Osteoporotic Fracture

**Fig. 1:**
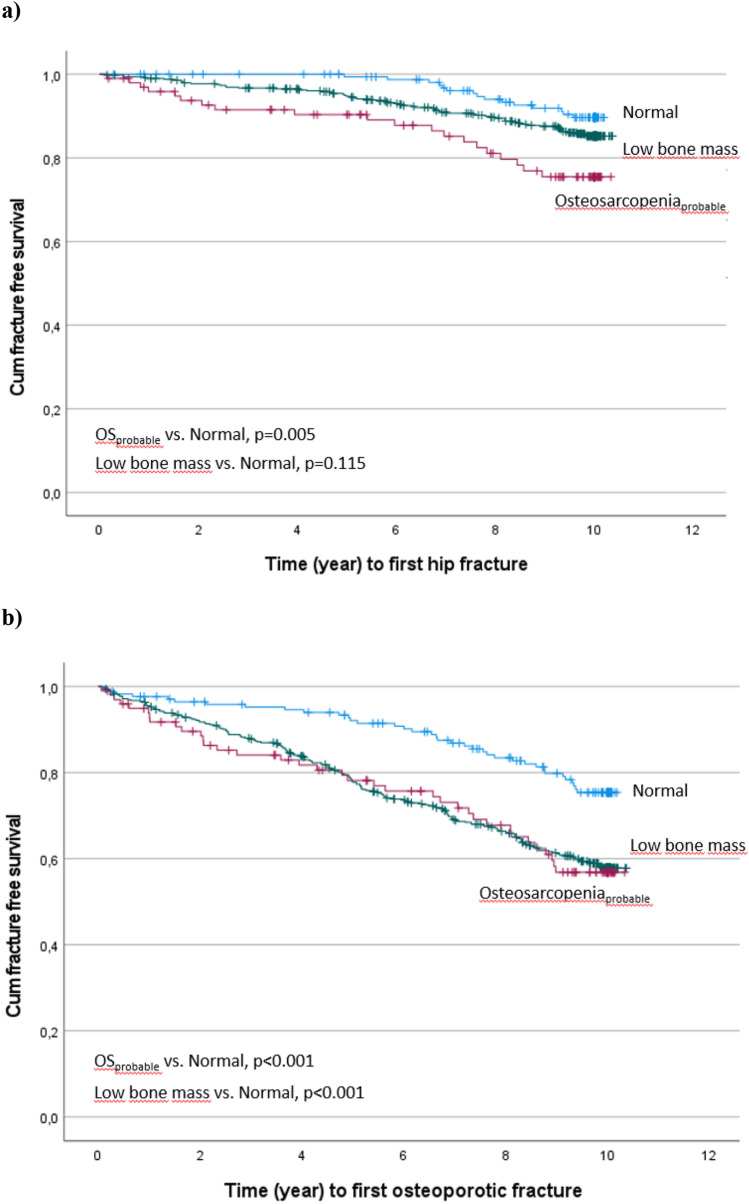
10-year fracture-free survival for (**a**) hip and (**b**) major osteoporotic fractures based on categorization as normal, low bone mass, or probable osteosarcopenia at baseline. Cumulative fractions of women without fracture are shown . *p*-values (log-rank test) are reported (reference category, normal). End point was date of first fracture (or end of follow-up time, if fracture free) and death date as censor

The 10-year risk of hip fracture was greater in the osteosarcopenia_probable_ group compared to the normal group (HR 2.89 [1.48–5.65]) even after adjustment (HR 2.67 [1.34–5.32]) (Table 2) and higher than in the LBM group (p < 0.016). Mirroring this, time to first hip fracture differed between groups (*p* = 0.005) (Fig. 1a); thus, first hip fracture occurred 0.3 year earlier in the osteosarcopenia_probable_ group than in the LBM group and 1.9 years earlier than in the normal group. The median time to fracture was 5.7, 6.0, and 7.6 years in the respective groups.

**Table 2:** 10-year risk for first incident hip and major osteoporotic fracture and 10-year mortality risk in the probable osteosarcopenia and low bone mass groups. The low bone mass group is compared to the normal reference and to probable osteosarcopenia

	OS_prob_		Low Bone Mass		Low Bone Mass	
	(ref normal)		(ref normal)		(ref OS_prob_)	
	HR [95% CI]	*p*-value	HR [95% CI]	*p*-value	HR [95% CI]	*p*-value
1st incident fracture						
Hip						
Unadjusted	2.89 [1.48–5.65]	**0.002**	1.56 [0.89–2.74]	0.119	0.54 [0.33–0.89]	**0.016**
Adjusted^*^	2.67 [1.34–5.32]	**0.005**	1.62 [0.92–2.85]	0.094	0.61 [0.36–1.03]	0.064
Major Osteoporotic						
Unadjusted	2.13 [1.35–3.38]	**0.001**	2.05 [1.44–2.91]	** < 0.001**	0.96 [0.67–1.37]	0.819
Adjusted^*^	2.04 [1.27–3.27]	**0.003**	2.08 [1.46–2.97]	** < 0.001**	1.02 [0.70–1.48]	0.911
Mortality						
10-year mortality						
Unadjusted	2.26 [1.46–3.51]	** < 0.001**	1.07 [0.75–1.55]	0.703	0.48 [0.334–0.675]	** < 0.001**
Adjusted^**^	1.91 [1.21–3.04]	**0.006**	0.94 [0.64–1.38]	0.76	0.49 [0.339–0.714]	** < 0.001**

Bold values indicate statistical significance (p < 0.05)

Hazard Ratio (HR) using Cox regression. Probable osteosarcopenia (low bone mass plus confirmed sarcopenia or probable sarcopenia)

^*^Adjusted for vitamin-D, alcohol, and polypharmacy (≥ 3 medications)

^*^^*^Adjusted for smoking, alcohol, polypharmacy, albumin, and CRP

For major osteoporotic fracture, the risk was elevated for both the osteosarcopenia_probable_ (HR 2.13 [1.35–3.38]) and the LBM group (HR 2.05 [1.44–2.91]) and withstood adjustment (Table 2). Illustrating this, time to first osteoporotic fracture differed from the normal reference group (p < 0.001 for both) (Fig. 1b). In the osteosarcopenia_probable_ group, the first osteoporotic fracture occurred within the shortest time frame and 2.1 years earlier than in the normal group. This was also earlier than in the LBM group, at 0.3 years. The median time to fracture was 4.5, 4.8, and 6.6 years in the respective groups.

An overview of major osteoporotic fractures distributed by site is reported in Supplementary Table 2**.**

### Risk of Death

**Fig. 2:**
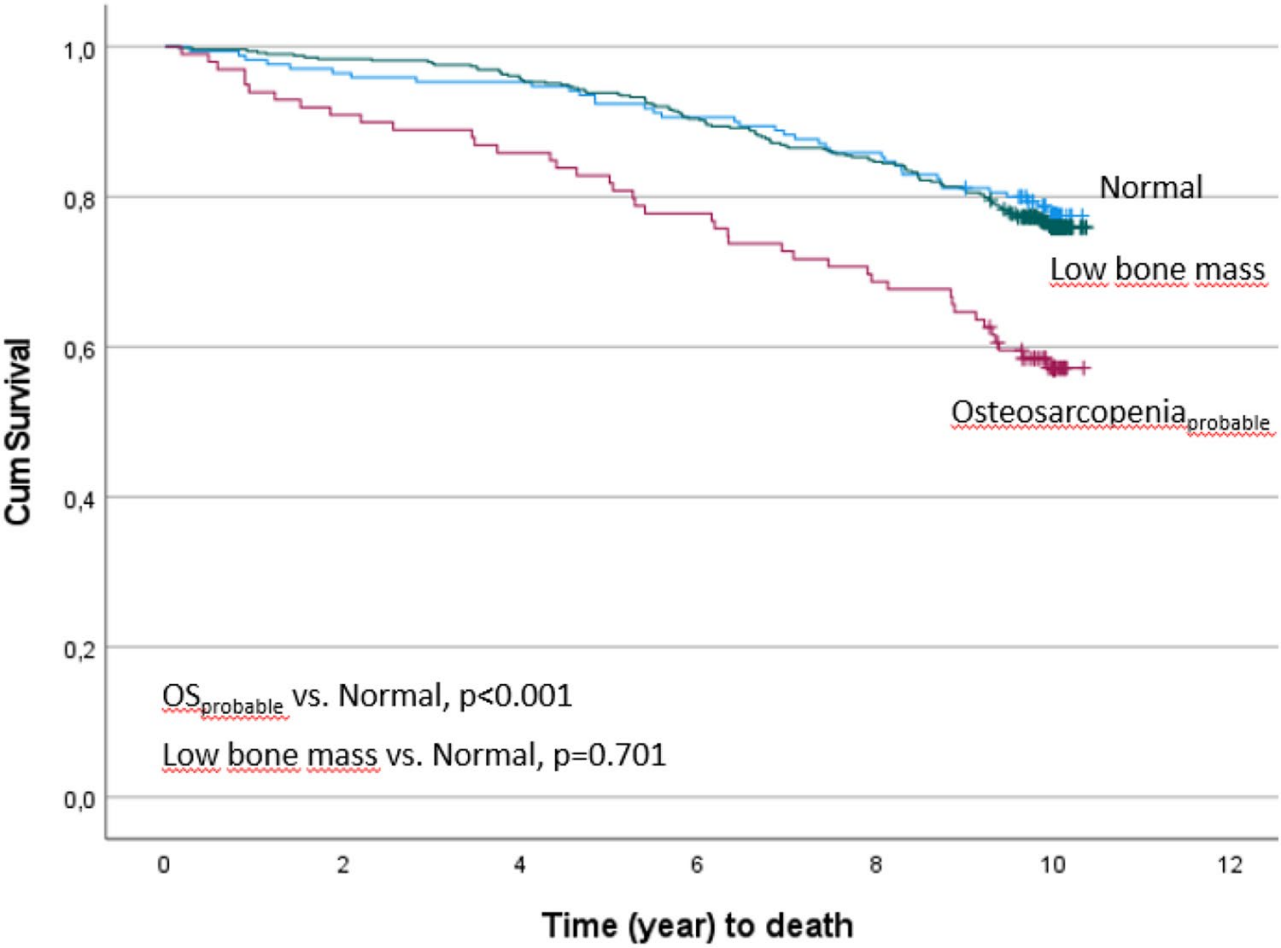
10-year survival based on categorisation as normal, low bone mass, or probable osteosarcopenia at baseline. *p*-values (log-rank test) are reported (reference category, normal)

Incident number of deaths was highest in the osteosarcopenia_probable_ group (*n* = 42 (42.4%), LBM *n* = 117 (23.9%), normal *n* = 38 (22.4%)). The osteosarcopenia_probable_ group had a greater 10-year risk of mortality compared to the normal group (HR 2.26 [1.46–3.51]), even after adjustment, while the LBM group did not (HR 1.07 [0.75–1.55]) (Table 2). As demonstrated in Fig. 2, women in the LBM group had half the risk of dying than the osteosarcopenia_probable_ group (HR 0.48 [0.33–0.68]).

## Discussion

This study contributes to the awareness of musculoskeletal health and the impact on the aging person. In a community-dwelling, population-based cohort of older women, we show that probable osteosarcopenia clearly confers a higher overall risk of fracture and mortality more so even than low bone mass alone.

In this cohort, at age 75 the proportion of women with confirmed osteosarcopenia is three percent, which is comparable to a few other population-based studies of northern European women [10, 11]. Establishing an accurate estimate in community-dwelling individuals is difficult, since osteosarcopenia is related to age and sex and on the definitions used [8, 9, 15].

Two studies, using the same definition of sarcopenia as us, one Danish cross-sectional (*n* = 529, mean age 75; range 65–93) and one British longitudinal study (*n* = 405, median age 76) reported prevalence at 1.5% and 2%, respectively [10, 11]. This is most likely explained by the inclusion of both sexes, as the prevalence of osteosarcopenia has been reported higher among older women than men [2, 33].

Another longitudinal study (*n* = 1114 women, mean age 77.6, different ethnicities) reported prevalence of osteosarcopenia to be 12% [18]. The more than double prevalence compared to our study can, at least partly, be explained by the use of the original (EWGSOP1) criteria of sarcopenia, which have been shown to give a higher prevalence of osteosarcopenia compared to EWGSOP2 [34]. This emphasizes the effect of how osteosarcopenia is defined; in an Australian study (*n* = 1032), despite a lower mean age (62 years) the reported prevalence was 8.3–9.7% [13], when including low muscle mass or low muscle strength alone.

Prevalence of confirmed osteosarcopenia increases with age, in our cohort from 3% at 75 to ~ 9% at 85y (although these are the most conservative assessment – when we also consider those with probable osteosarcopenia, an estimated two to four times more women may be affected).

The higher prevalence from using the EWGSOP1 sarcopenia criteria was also obvious in a longitudinal study by Salech et al. with prevalence starting at 8.9% (age 60–69.9), rising to 18.3% (age 70–79.9), and 33.7% (> 80 years) [9]. However, the lower prevalence in our study may, at least partially, reflect the relatively good health of the cohort. While this may introduce a ‘healthy cohort bias,’ on the other hand, due to the unique study design whereby all participants are identically aged we are likely seeing a more “true” age-related prevalence of osteosarcopenia in older, community-dwelling women.

The relationship between bone and muscle is closely associated throughout life [35]. In the OPRA cohort all women with sarcopenia had low bone mass, while conversely only a small percentage (4%) of those with low bone mass had sarcopenia, as has also been noted in other studies [14]. Furthermore, osteoporosis is more prevalent among women with sarcopenia than among those in the low bone mass [10, 14].

Women in both the probable osteosarcopenia group and in the low bone mass group had a two-time greater risk of major osteoporotic fracture compared to women in the normal group. However, most importantly women with probable osteosarcopenia had a higher risk of hip fracture even compared to those with low bone mass alone. This is reflected in the clinical characteristics of women with probable osteosarcopenia, who were frailer and also had more falls than women with low bone mass [12, 14, 18, 33], indicating the need to consider sarcopenia (not just confirmed, but also probable) as part of fracture management.

Interestingly, the low bone mass group had more incident vertebral, proximal humerus, and distal radius fractures but fewer hip fractures than the probable osteosarcopenia group. A finding that might indicate a better physical capability to react to a fall. Hip fracture occurs earlier in those with probable osteosarcopenia and there appears to be a higher imminent risk of fracture based on the survival curves. Although the difference in median time to first hip and major osteoporotic fracture between probable osteosarcopenia and low bone mass group was less than half a year, this period of “gained” independence is of great importance in old age.

Our results on osteosarcopenia and fracture aligns with other longitudinal studies, although our risk estimates are higher [9, 13, 15, 18]. One with 5,640 person-years of follow-up reported an increased risk of fracture (HR 1.54 [1.13–2.08]) [9]. Studies of different designs, mixed sexes and lower ages, report similarly increased 10- or 5-year risk of fractures with osteosarcopenia but not compared to low bone mass or sarcopenia alone [13, 15], which draws attention to the difficulties in comparing studies.

Similar to our observations, other longitudinal studies report higher mortality risk with osteosarcopenia but not low bone mass or sarcopenia alone. But again, direct comparison is difficult because of younger and mixed populations and definitions [9, 13]. The higher mortality is not surprising, since these women have lower BMI, are weaker, more physically limited, have poorer kidney function, a less healthy lifestyle, and take more medications (an expression of co-morbidity). In short, these women are frailer, and this geriatric syndrome has undoubtedly proven to be related to increased mortality [36].

Taken together the data highlights that in older women, as important as screening for and treating low bone mass is the evaluation of muscle parameters and a focus on fall prevention in order to maintain musculoskeletal integrity. Illustrating this, we have previously shown that within a group categorized as ‘low risk’ based on FRAX score, those who are frail actually have a high risk of fracture [37]. While there is no current treatment for osteosarcopenia, interventions such as resistance training and anti-osteoporosis treatment [38] could directly or indirectly prevent further deterioration of muscle mass and strength that often follows fractures, particularly hip fracture.

### Strengths and Limitations

Strengths of the study include the longitudinal, population-based design which allows for generalization to the target population. Uniquely, the women were all the same age allowing assessment of changes in prevalence and outcomes over time; giving a perspective on “chronological” and “biological” trajectory of change in musculoskeletal aging. Age and 10-year follow-up give the opportunity to investigate osteosarcopenia during a critical period in life where a decline in health with falls, fractures, and mortality are more likely and therefore provide important information on when and how to be more attentive to this disease. *Second*, all individuals were Caucasian, the same gender and age, therefore reducing confounding related to sex hormones, accumulation of co-morbidities, and ethnicity. *Third*, the participation rate was high (65%, 75%, and 76% at respective visits) and the cohort constituted as much as 33% of all 75-year-old women living in Malmö, Sweden at the time of inclusion. *Fourth*, this study includes the most used criteria of both osteopenia/osteoporosis (WHO) and sarcopenia (EWGSOP2) to define osteosarcopenia. Besides, EWGSOP2 is the preferred and most cited definition in Europe and Australia [10]. *Fifth*, in contrast to other studies, both overall fracture risk as well as risk of hip fracture were investigated; and characterization of all types of osteoporotic fractures in relation to the three comparative groups provided important practical information on possible mechanisms leading to fractures. In other studies, fractures were mostly self-reported or lacked detailed information on collection. Only one longitudinal, population-based study provided information on fracture type.

Study limitations are also acknowledged. *First*, we adopted the cut-offs from the EWGSOP2 definition and, while recognizing that locally derived reference data might have been useful, the advantage lies in facilitating comparison with the existing literature. *Second*, it may have been preferable to use the same osteosarcopenia classification for estimating both prevalence and its associated risks. However, we used the strictest meaning (‘confirmed osteosarcopenia’) to most accurately estimate prevalence of the condition among older community-dwelling women. For the risk analyses we included both ‘confirmed’ and ‘probable’ osteosarcopenia in the definition partly due to the low number of women with confirmed sarcopenia but also because low muscle strength (sufficient to assign probable osteosarcopenia) is considered the most important influence on clinical outcome [39, 40]. We assume that the observed elevated mortality risk would also be apparent in those with more severe muscle loss, given the overlap between sarcopenia, osteosarcopenia, and frailty [2, 41]. *Third*, when calculating the prevalence of osteosarcopenia, two or three variables were required (T-score and muscle strength, as well as muscle mass for ‘confirmed’ osteosarcopenia), which might lead to sampling bias. *Fourth*, the OPRA cohort did not have sufficiently detailed information about co-morbidities to create a co-morbidity index. Hence, as a proxy we adjusted for polypharmacy, which could be considered a limitation, although it is reported to be correlated with co-morbidity. In addition, while we know that some have used glucocorticoids ‘at some point,’ detailed information on date, duration, or dose was not available. Since glucocorticoid use was overall very low (*n* = 29 at age 75; *n* = 41 at age 80; *n* = 22 at age 85), we have not corrected for this. *Fifth*, those who chose not to participate might have worse health, but this is common in most studies of older populations [42] and might explain the low number with sarcopenia in the cohort. *Sixth*, knee strength was used since handgrip strength was not available at baseline. Although evidence for association between handgrip and knee strength in older individuals is conflicting [43], isometric torque method of the lower limb is a validated tool for determination of overall muscle strength [16]. And although handgrip strength is commonly used to predict physical function, knee strength might be a more appropriate proxy since there is a greater age-related loss of leg strength compared to arm strength [44, 45]. This could possibly relate to a higher degree of disuse of the lower extremities with age.

## Conclusion

In this longitudinal study, confirmed osteosarcopenia prevalence increased from 3.0% at age 75 to 9.2% at age 85. Women with probable osteosarcopenia had significantly increased risk of hip and major osteoporotic fractures and mortality. Probable osteosarcopenia was also associated with higher frailty and lower physical functioning. The addition of sarcopenia to low bone mass markedly increases the risk of both fracture and mortality. To summarize, in older women the clinical approach should focus on the musculoskeletal system as a whole; in addition to screening for low bone mass, screening for, or at least being aware that low muscle strength is an additional clinical risk factor, could also be valuable.

### Supplementary Information

Below is the link to the electronic supplementary material.

Supplementary file1 (DOCX 24 KB)
