# Supplementary material for: Osteosarcopenia: Prevalence and 10-Year Fracture and Mortality Risk – A Longitudinal, Population-Based Study of 75-Year-Old Women
Source: Calcif Tissue Int. 2024 Feb 1;114(4):315–25. doi: 10.1007/s00223-023-01181-1 (PMC10957698; doi:10.1007/s00223-023-01181-1)
Supplement: Supplementary file 1 — Supplementary file1 (DOCX 24 KB) [file 223_2023_1181_MOESM1_ESM.docx]

# Osteosarcopenia - Prevalence and 10-year Fracture and Mortality Risk: A Longitudinal, Population-based Study of 75-year-old Women

Tine Kolenda Paulin^1 2^, Linnea Malmgren^1 2^, Fiona E. McGuigan^1^, Kristina E. Åkesson^1 3^

**Affiliation of authors**

^1^ Clinical and Molecular Osteoporosis Research Unit, Department of Clinical Sciences, Lund University, Malmö, Sweden

^2^ Department of Geriatrics, Skåne University Hospital, Malmö, Sweden

^3^ Department of Orthopaedics, Skåne University Hospital, Malmö, Sweden

**Corresponding author**

Tine Kolenda Paulin, MD

Department of Clinical Sciences Malmö, Lund University

Department of Geriatrics, Skåne University Hospital

Clinical Research Centre,

Jan Waldenströms gata 35, 214 28 Malmö, Sweden

Telephone: 040 391131

E-mail: [tine.kolendapaulin@skane.se](mailto:tine.kolendapaulin@skane.se)

ORCHID: 0000-0002-7248-2534

**Supplementary Table 1**.

Phenotype combinations and the categorisation into groups: 1) Normal, 2) Low bone mass and 3) Probable osteosarcopenia

| **Combinations Possible** | | |  |  |  |
| --- | --- | --- | --- | --- | --- |
| **BMD** | **Muscle Strength** | **Muscle**  **Mass** | **n** | **Group description** | n |
|  |  |  |  |  |  |
| Normal | Normal | Normal | 170 | Normal | 170 |
|  |  |  |  |  |  |
| Low | Normal | Normal | 489 | Low Bone Mass | 489 |
|  |  |  |  |  |  |
| Low | Low | Normal | 70 | Osteosarcopenia _probable_ | 99 |
| Low | Low | Low | 29 |  |  |
|  |  |  |  |  |  |
| Normal | Low | Low | 0 | Not included in the analyses | 96 |
| Normal | Low | Normal | 9 |  |  |
| Normal | Normal | Low | 8 |  |  |
| Low | Normal | Low | 79 |  |  |
|  |  |  |  |  |  |
| -- | -- | -- | 116 | Data not available | 116 |
|  |  |  |  |  |  |

*Probable osteosarcopenia (low bone mass plus confirmed sarcopenia or probable sarcopenia)*

**Supplementary Table 2**.

Incident first major osteoporotic fracture (i.e. number of women having their first osteoporotic fracture of any site) and first specific fracture by site during 10-year follow-up.

|  | **All women**  *N=758* | **Normal**  *N=170* | **Low Bone Mass**  *N=489* | **Osteosarcopenia_probable_**  *N=99* |
| --- | --- | --- | --- | --- |
|  | N (%) | N (%) | N (%) | N (%) |
|  |  |  |  |  |
| **First major osteoporotic fracture** | 265 (35.0) | 37 (21.8) | 192 (39.3 | 36 (36.4) |
|  |  |  |  |  |
| **First fracture by site** |  |  |  |  |
| Hip | 100 (13.2) | 15 (8.8) | 65 (13.3) | 20 (20.2) |
| Vertebral | 111 (14.6) | 12 (7.1) | 86 (17.6) | 13 (13.1) |
| Pelvic | 28 (3.7) | 4 (2.4) | 22 (4.5) | 2 (2.0) |
| Prox humerus | 49 (6.5) | 8 (4.7) | 37 (7.6) | 4 (4.0) |
| Distal radius | 78 (10.3) | 8 (4.7) | 59 (12.1) | 11 (11.1) |
|  |  |  |  |  |
